# Study of the mandibular canal and its surrounding canals by multi-view cone-beam computed tomography

**DOI:** 10.1186/s13244-024-01676-x

**Published:** 2024-04-08

**Authors:** Yingqi Chen, Qianting Liao, Yuexia Wu, Rui He, Nanxiang Zhang, Yanfen Liao, Keqian Lian

**Affiliations:** 1 Department of Stomatology, The First Affiliated Hospital of Sun Yat-Sen University, Guangzhou, 510080 China; 2 Stomatological Hospital, Southern Medical University, Guangzhou, 510280 Guangdong China; 3 Department of Medical Statistics, School of Public Health, Sun Yat-Sen University, Guangzhou, 511400 China; 4 Department of Stomatology, The Second People’s Hospital of Panyu Guangzhou, Guangzhou, 511400 China

**Keywords:** Anatomy, Cone-beam computed tomography, Lingual canal, Mandible, Mandible canal

## Abstract

**Objectives:**

To determine the mandibular anatomical structures by observing cone-beam computed tomography (CBCT) from multiple angles.

**Materials and methods:**

This retrospective study analyzed 1593 consecutive CBCT images. Ultimately, 95 CBCTs met the inclusion criteria. The mandibular, inferior lingual, and bony canals at the tooth apex were studied by multi-angle observation CBCT. Descriptive statistics were used for statistical analysis.

**Results:**

It is beneficial to further observe the anastomosis of the mandibular, lingual, and mandibular canals when the course of the mandibular lingual canal is observed on CBCT cross-section. The frequency of the inferior lingual canal anastomosis with the mandibular canal was 43.2% (95% confidence interval (CI) 33, 53.3) in the sample. The mental foramen was located below the long axis of the tooth in a few samples, with an occurrence rate of 29.5% (95% CI 20.1, 38.8). The occurrence rate of various types of the bony canal at the apex of the tooth in canines, first premolars, second premolars, first molars, and second molars under the root apex was recorded through the multi-angle observation of the dental volume reformat (DVR) and three-dimensional (3D) levels in CBCT.

**Conclusion:**

This study demonstrates the utility of CBCT imaging in examining mandibular anatomy from multiple angles, providing valuable insights into anatomical variations, and enhancing our understanding of mandibular structures. This research emphasizes the crucial role of meticulous CBCT examination in precisely identifying and understanding key anatomical structures, ultimately reducing the risk of surgical complications.

**Critical relevance statement:**

By examining cone-beam computed tomography scans from various perspectives, it is possible to determine the precise position of anatomical structures within the jaw. This allows for a more accurate assessment, reducing the risk of harm to these structures during treatment.

**Key points:**

• It is crucial to utilize image data effectively to enhance the comprehension of human anatomy.

• We captured detailed images of the mandible from different angles and orientations utilizing cone-beam computed tomography (CBCT).

• This study provides essential anatomical information for procedural planning to ensure optimal outcomes and patient safety.

**Graphical Abstract:**

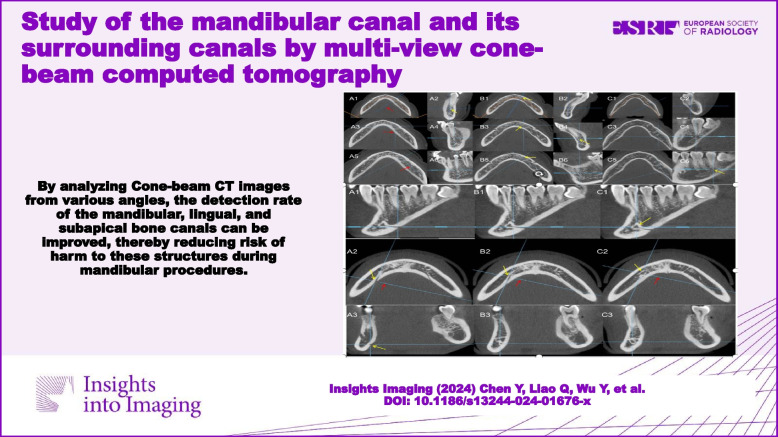

**Supplementary Information:**

The online version contains supplementary material available at 10.1186/s13244-024-01676-x.

## Introduction

The mandibular nerve canal (MC) and its surrounding canal conformation are highly significant for implant placement, mandibular surgeries, and root canal treatments [1–3]. Another important channel in the mandible is the mandibular lingual canals (LCS). Severe damage to the mandibular lingual canal can lead to asphyxiation and death [4, 5]. Some studies have shown anastomosis of the mandibular lingual canals (LCS) and MC in the mandible; however, the specific situation and direction of the anastomosis between the MC and LCS have been sparsely reported. Additionally, other canals around the MC, such as the bony canal at the apex of the tooth (BCAT), have also been less reported [6].

Rapid and accurate identification of the MC and its relationship to surrounding canals will further enhance our understanding of the blood vessel distribution in the mandible. The follow-up will be beneficial to the subsequent use of artificial intelligence to automatically identify the MC and its surrounding canals, thereby greatly reducing the damage to these canals during the clinical process.

Cone-beam computed tomography (CBCT) is widely used in the oral cavity, providing convenience for MC and LCS detection. Therefore, this study will further assess the MC, LCS, and BCAT by observing CBCT from multiple angles, clarifying the anatomical characteristics of the MC, anastomosis of the lower lingual foramen and MC, and anatomical features of the dental canal.

## Materials and methods

**Fig. 1:**
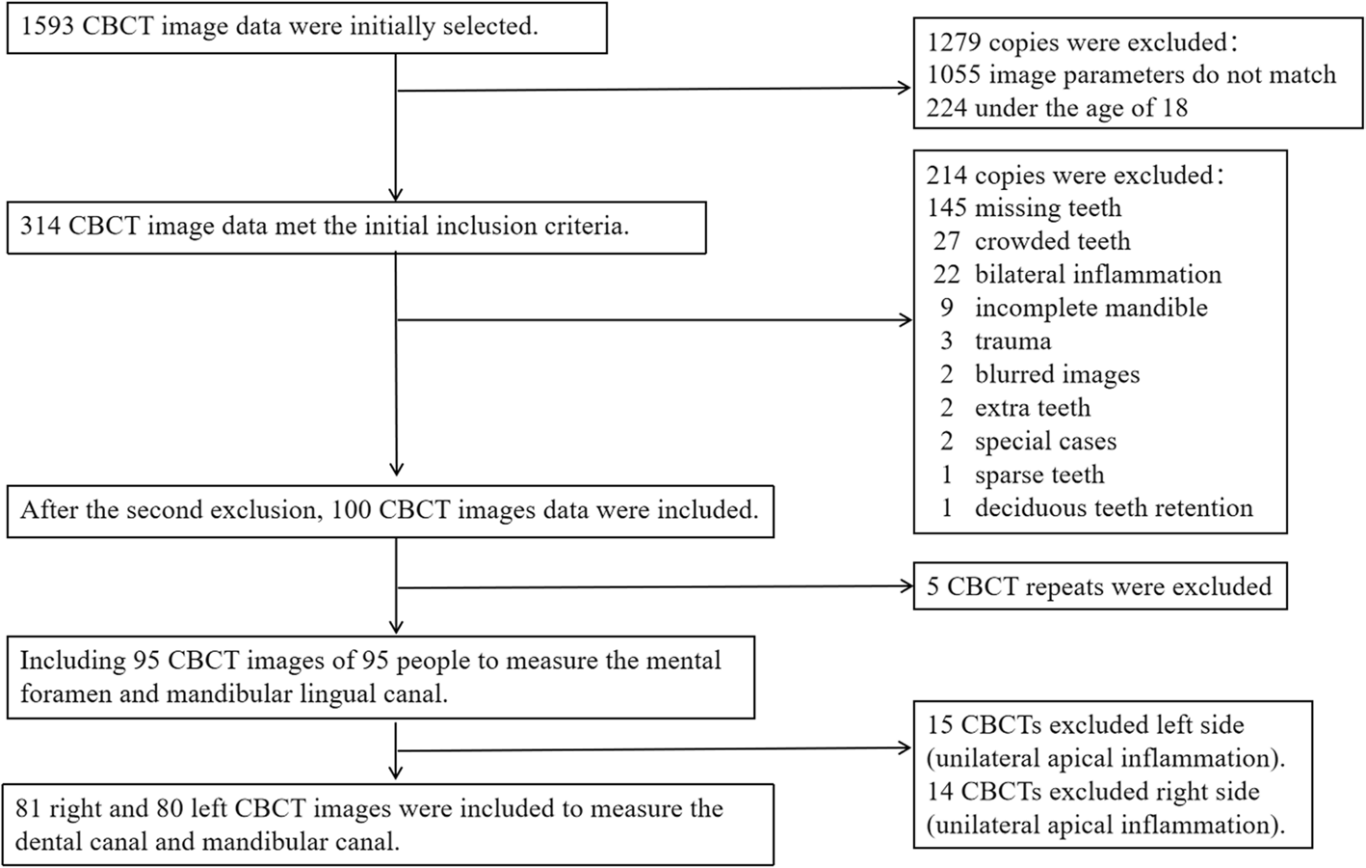
Flow chart for sample selection. During the sample inclusion process, a total of 95 samples were included after meeting the exclusion criteria. Out of these, 81 samples were taken from the right side and 80 samples were taken from the left side to measure the subapical bone canal and mandibular canal. The dental canal shown in the picture refers to the bony canal at the tooth apex

A total of 1593 CBCT images recorded continuously in the Photograph Room of the Department of Stomatology, the First Affiliated Hospital of Sun Yat-sen University, China, were selected for this study from January 2021 to March 2022. According to the exclusion process in Fig. 1, 95 CBCT images of 95 individuals were included to measure the mental foramen and LCS. Among them, there were 42 men and 53 women, with an average age of 36.7 years. In addition, 81 right and 80 left CBCT images were included to measure the BCAT and MC. Moreover, when calculating the statistical significance related to the BCAT, samples with unilateral apical inflammation were excluded, and CBCT samples were included.

The KaVo group (model: i-CATFLX) image acquisition system and 3D cephalometric mode were used with a high resolution and of volume 16 D × 13 H or 16 D × 10 H, voxel 0.25 mm, scanning time 26.9 s, exposure time 7.4 s, tube voltage 120 kV, and tube current 5 mA. All image analysis uses the OnDemand3D application software system uniformly, and the images are carefully read under standardized viewing conditions.

**Fig. 2:**
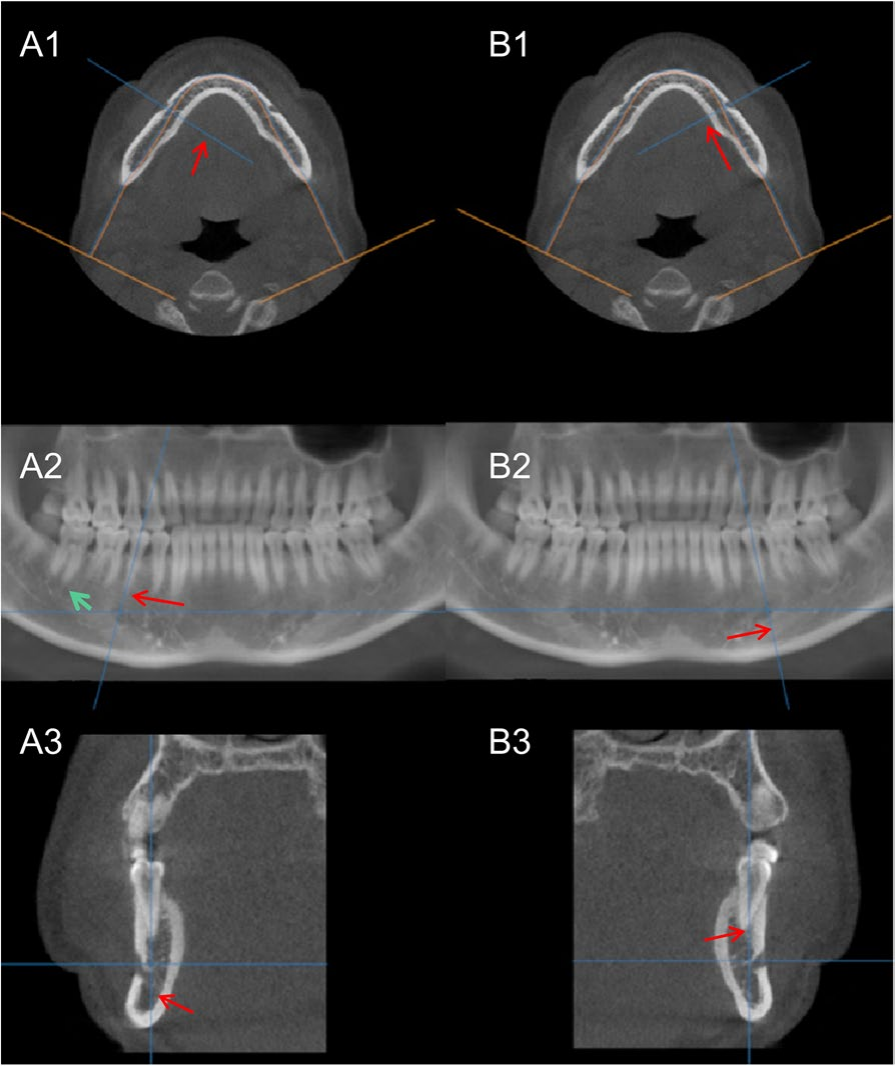
At the DVR level, it will be more accurate to determine the relationship between the mental foramen and tooth position by observing the long axis direction of the tooth. **A1, A2, A3** Right side. **B1, B2, B3** Left side. **A1, B1** Cross-section in the front-back direction of the DVR. **A2, B2** Panoramic image at the DVR. **A3, B3** Axial plane of the DVR. **A2** White dense image is the bony canal at the tooth apex (green arrow). The mental foramen is observed by moving the observation axis back and forth in the direction of the long axis of the tooth at the DVR level

In the dynamic voltage restorer （DVR） interface, observe the visibility of the upper and lower walls of the MC from the angle of the long axis of the tooth. Measurements included the height of the MC in the cranial and caudal direction (Height1), the width of the MC in the buccal and lingual direction (Width1), the distance from the lower wall of the MC to the root apex of the tooth (Height2) (multiple teeth considered for mesial roots), the distance from the lower wall of the MC to the lower edge of the mandible (Height3), the distance from the upper wall of the MC to the apex of the corresponding tooth root (Height4), the horizontal distance from the buccal wall of the MC to the buccal bone plate (Width2), and the horizontal distance from the lingual wall of the MC to the lingual bone plate (Width3) (Fig. 2).

**Fig. 3:**
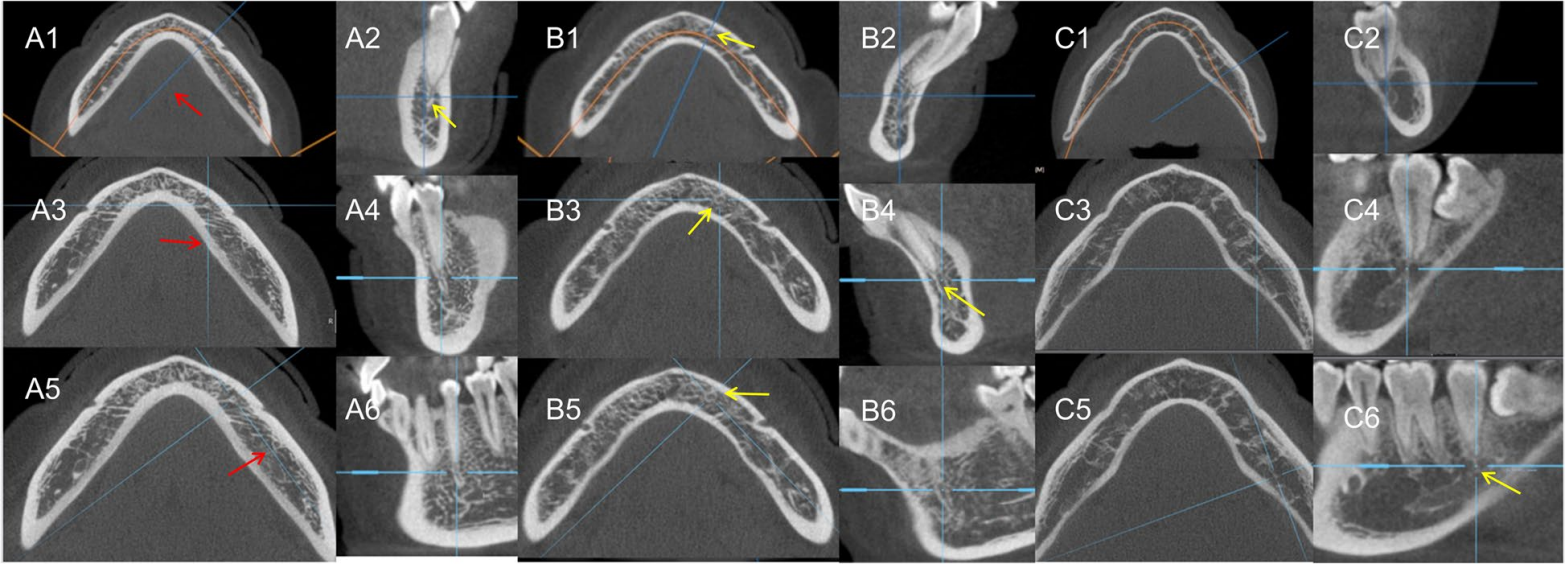
The same bony canal at the tooth apex can be observed on different surfaces. **A1, B1, C1** Axial plane of the DVR. **A2, B2, C2** Cross-section of the DVR. **A3, B3, C3/A5, B5, C5** axial plane of the 3D. **A4, B4, C4/A6, B6, C6** Sagittal plane of the 3D. The blue axis pointed by the red arrow in figures **A3** and **A5**. By rotating the axis horizontally, the bony canal at the tooth apex (yellow arrow) can be observed from different angles. Pictures A, B, and C are from different tooth positions. Observed in the same way

The appearance of BCAT, the maximum width of the bone canal at the two interfaces, and anastomosis between the BCAT and MC on both the DVR and 3D surfaces (including the normal 3D angle and profile) were recorded (Fig. 3). The appearance of white dense images below the BCAT on the DVR interface was recorded (Fig. 2A2 (green arrow)). Owing to the different conditions of the BCAT in the mandible, we divided them into the following three main types: type 1—a clear bone canal directly connected to the apex and running below the apex (Fig. 3A); type 2—between the above two cases, it is directly connected with the apex of the apex and runs below the apex (picture 3B); and type 3—directly connected to the apex of the apex with the white line running below (Fig. 3C).

**Fig. 4:**
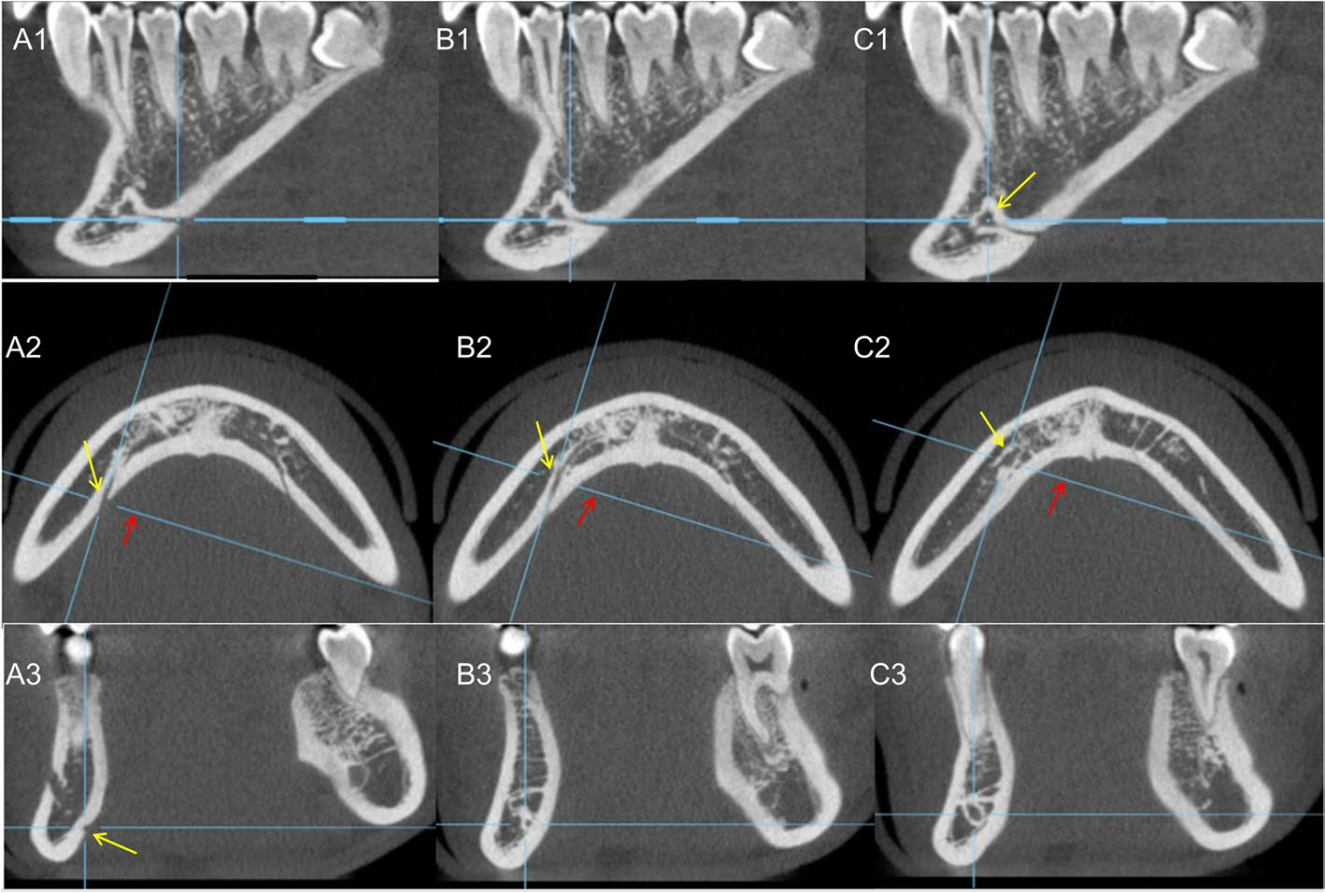
The mandibular lingual canal distributed laterally enters the mandible and merges with the mandibular canal. **A1, B1, C1** Sagittal plane of the 3D. **A2, B2, C2** Axial plane of the 3D. **A3, B3, C3** Coronal plane of the 3D. After observing the lateral lingual canal on the axial plane, adjust the observation axis and move it (red arrow) forward and backward to observe the anastomosis between the lateral lingual canal and the mandibular canal (yellow arrow)

In the 3D interface, the path of the LCS is tracked by observing the normal 3D surface and the same angle as the transverse direction of the LCS (Fig. 4), further clarifying the alignment between LCS and MC. The tooth position when the lingual foramen enters the mandible and coincides with the MC was recorded. The position of the anastomosis is related to the proximal and distal directions of the mental foramen and upper and lower (head and tail) directions were also recorded.

### Statistical analysis

Statistical analysis was performed using the SPSS 25.0 software. The relationship between binary and numerical variables was tested using an independent samples *t*-test. We also calculated their 95% confidence interval.

## Results

### Mandibular canal and mental foramen

Under the DVR level observation, the measurement data of the MC in the mandibular bilateral canines, premolars, and molars include the following content: the visibility rate of the upper and lower canal walls and height of the canal in the cranial-caudal direction (Height 1), the width of the canal in the buccal and lingual directions (Width 1), the vertical distance from the canal wall below the canal to the tooth apex (Height 2), the vertical distance from the canal wall below the canal to the lower edge of the mandible (Height 3), the distance from the canal wall above the canal to the tooth apex (Height 4), the horizontal distance from the buccal canal wall to the buccal bone plate (Width 2), and the horizontal distance from the lingual canal wall to the lingual bone plate (Width 3) (Table 1). Additionally, the rate of anastomosis between the MC and subapical bony canal was recorded at the DVR level (Table 2). An anastomosis reference image is illustrated in Fig. 3C2, C4, C6. The anastomosis rate of the MC to LCS was recorded in 3D (Table 3).

**Table 1:** Assessment of the MC

	47	46	45	44	43	33	34	35	36	37
Clear upper wall (%)	79/81 (97.5)	70/81 (86.4)	70/81 (86.4)	58/81 (71.6)	36/81 (44.4)	29/80 (36.2)	55/80 (68.8)	71/80 (88.8)	70/80 (87.5)	77/80 (96.2)
Clear lower wall (%)	79/81 (97.5)	70/81 (86.4)	70/81 (86.4)	58/81 (71.6)	36/81 (44.4)	29/80 (36.2)	55/80 (68.8)	71/80 (88.8)	70/80 (87.5)	77/80 (96.2)
Height 1 ± SD	2.70 ± 0.55	2.40 ± 0.45	3.55 ± 1.73	1.75 ± 0.74	1.19 ± 0.51	1.17 ± 0.29	1.85 ± 0.88	3.31 ± 1.58	2.51 ± 0.44	2.69 ± 0.48
95% CI	2.58, 2.82	2.29, 2.51	3.14, 3.96	1.56, 1.94	1.02, 1.36	1.06, 1.28	1.62, 2.08	2.94, 3.68	2.41, 2.61	2.58, 2.8
Width 1 ± SD	2.28 ± 0.45	2.08 ± 0.41	3.32 ± 1.74	1.73 ± 0.71	1.12 ± 0.53	1.25 ± 0.44	1.73 ± 0.85	2.95 ± 1.47	2.16 ± 0.41	2.31 ± 0.34
95% CI	2.18, 2.38	1.98, 2.18	2.91, 3.73	1.55, 1.91	0.95, 1.29	1.09, 1.41	1.51, 1.95	2.61, 3.29	2.06, 2.26	2.23, 2.39
Height 2 ± SD	7.19 ± 2.56	9.80 ± 2.43	8.67 ± 2.76	8.76 ± 2.85	9.07 ± 3.47	9.54 ± 2.94	8.87 ± 2.94	8.78 ± 2.95	9.87 ± 2.71	7.21 ± 2.46
95% CI	6.63, 7.75	9.23, 10.37	8.02, 9.32	8.03, 9.49	7.94, 10.2	8.47, 10.61	8.09, 9.65	8.09, 9.47	9.24, 10.5	6.66, 7.76
Height 3 ± SD	8.72 ± 1.88	8.12 ± 1.62	9.07 ± 1.71	0.35 ± 1.65	9.25 ± 1.94	8.72 ± 1.69	9.95 ± 1.48	9.09 ± 2.12	7.99 ± 1.72	8.76 ± 2.16
95% CI	8.31, 9.13	7.74, 8.5	8.67, 9.47	9.93, 10.77	8.62, 9.88	8.1, 9.34	9.56, 10.34	8.6, 9.58	7.59, 8.39	8.28, 9.24
Height 4 ± SD	4.49 ± 2.63	7.39 ± 2.41	5.12 ± 3.05	7.02 ± 2.83	7.89 ± 3.50	8.37 ± 2.89	7.02 ± 2.92	5.47 ± 3.29	7.35 ± 2.66	4.52 ± 2.43
95% CI	3.91, 5.07	6.83, 7.95	4.41, 5.83	6.29, 7.75	6.75, 9.03	7.32, 9.42	6.25, 7.79	4.7, 6.24	6.73, 7.97	3.98, 5.06
Width 2 ± SD	7.19 ± 1.21	6.13 ± 1.15	3.33 ± 2.33	3.02 ± 0.98	4.00 ± 1.23	4.60 ± 1.11	3.20 ± 0.96	3.54 ± 2.20	6.04 ± 1.09	7.15 ± 1.31
95% CI	6.92, 7.46	5.86, 6.4	2.78, 3.88	2.77, 3.27	3.6, 4.4	4.2, 5	2.95, 3.45	3.03, 4.05	5.78, 6.3	6.86, 7.44
Width 3 ± SD	2.66 ± 0.81	2.83 ± 0.99	3.82 ± 1.54	5.16 ± 1.50	4.50 ± 1.57	4.74 ± 1.08	5.06 ± 1.29	3.92 ± 1.62	2.91 ± 1.07	2.66 ± 0.92
95% CI	2.48, 2.84	2.6, 3.06	3.46, 4.18	4.77, 5.55	3.99, 5.01	4.35, 5.13	4.72, 5.4	3.54, 4.3	2.66, 3.16	2.45, 2.87

*SD *Standard deviation, *CI *Confidence interval, *MC *Mandibular canal. The sample size is 81 on the right and 80 on the left

**Table 2:** Assessment of the BCAT

	47	46	45	44	43	33	34	35	36	37
*N*	12	38	32	23	27	26	26	28	30	15
**Occurrence rate of white dense images (%)**	0/N (0.0)	10/N (26.3)	1/N (3.1)	3/N (13.0)	3/N (11.1)	1/N (3.8)	4/N (15.4)	4/N (14.3)	10/N (33.3)	1/N (6.7)
**Occurrence rate DVR level (%)**	12/N (100.0)	38/N (100.0)	32/N (100.0)	21/N (91.3)	27/N (100.0)	26/N (100.0)	26/N (100.0)	28/N (100.0)	30/N (100.0)	15/N (100.0)
The diameter of the DVR level ± SD	0.65 ± 0.49	0.40 ± 0.28	0.21 ± 0.18	0.22 ± 0.17	0.43 ± 0.18	0.40 ± 0.19	0.28 ± 0.18	0.23 ± 0.21	0.36 ± 0.26	0.26 ± 0.27
95% CI	0.37, 0.93	0.31, 0.49	0.15, 0.27	0.15, 0.29	0.36, 0.5	0.33, 0.47	0.21, 0.35	0.15, 0.31	0.27, 0.45	0.12, 0.4
Connected to mandibular canal (%)	5/N (41.7)	11/N (28.9)	10/N (31.2)	3/N (13.0)	3/N (11.1)	0/N (0.0)	3/N (11.5)	8/N (28.6)	12/N (40.0)	6/N (40.0)
**Occurrence rate 3D level (%)**	12/N (100.0)	38/N (100.0)	32/N (100.0)	23/N (100.0)	27/N (100.0)	26/N (100.0)	26/N (100.0)	28/N 100.0)	30/N (100.0)	15/N (100.0)
The diameter of the 3D level ± SD	0.44 ± 0.17	0.36 ± 0.14	0.30 ± 0.18	0.35 ± 0.13	0.37 ± 0.16	0.42 ± 0.17	0.36 ± 0.21	0.28 ± 0.17	0.32 ± 0.23	0.25 ± 0.20
95% CI	0.34, 0.54	0.32, 0.4	0.24, 0.36	0.3, 0.4	0.31, 0.43	0.35, 0.49	0.28, 0.44	0.22, 0.34	0.24, 0.4	0.15, 0.35
Connected to mandibular canal (%)	8/N (66.7)	17/N (44.7)	9/N (28.1)	5/N (21.7)	3/N (11.1)	1/N (3.8)	8/N (30.8)	12/N (42.9)	23/N (76.7)	5/N (33.3)
3D normal surface occurrence rate (%)	12/N (100.0)	38/N (100.0)	32/N (100.0)	23/N (100.0)	26 /N (96.3)	25/N (96.2)	26/N (100.0)	28/N (100.0)	30/N (100.0)	14/N (93.3)
3D profile occurrence rate (%)	12/N (100.0)	37/N (97.4)	31/N (96.9)	23/N (100.0)	27/N (100.0)	25/N (96.2)	26/N (100.0)	28/N (100.0)	30/N (100.0)	14/N (93.3)

*N*, the total number of bony canal at the apex of the tooth in each tooth; *SD *Standard deviation, *CI *Confidence interval, *BCAT *The bony canal at the apex of the tooth, *DVR *Dental volume reformat, *3D *Three-dimensional. The sample size is 81 on the right and 80 on the left

**Table 3:** Frequency of the LCS and the MF

	Male	Female	Total
Left lingual canal anastomosis (95% CI)	31 (16.4, 45.5)	32.1 (19.1, 45.1)	31.6 (22.1, 41.1)
Right lingual canal anastomosis (95% CI)	31 (16.4, 45.5)	18.9 (8, 29.8)	24.2 (15.4, 33)
Lingual canal anastomosis (95% CI)	45.2 (29.5, 60.9)	41.5 (27.8, 55.2)	43.2 (33, 53.3)
Left double mental foramen (95% CI)	7.1 (-1, 15.3)	5.7 (-0.8, 12.1)	6.3 (1.3, 11.3)
Left double mental foramen (95% CI)	4.8 (-2, 11.5)	7.5 (0.2, 14.9)	6.3 (1.3, 11.3)
Double mental foramen (95% CI)	11.9 (1.7, 22.1)	13.2 (3.8, 22.6)	12.6 (5.8, 19.4)
Long axis of left mental foramen (95% CI)	16.7 (4.9, 28.4)	20.8 (9.5, 32)	18.9 (10.9, 27)
Long axis of right mental foramen (95% CI)	14.3 (3.2, 25.3)	22.6 (11, 34.3)	18.9 (10.9, 27)
Long axis of mental foramen (95% CI)	21.4 (8.5, 34.4)	35.8 (22.5, 49.2)	29.5 (20.1, 38.8)

*CI * Confidence interval, *LCS *Lingual canals, *MF *Mental foramen. The sample size is 95

The occurrence rate of double mental foramen bilaterally was 6.3%, and the total occurrence rate was 12.6%. The mental foramen occurs on the long axis of the tooth. The frequency of occurrence can be observed in Table 3 and the observation method in Fig. 2. The distribution of the mental foramina on the tooth position is primarily 45 (51/95), 44–45 (39/95), 45–46 (5/95), 35 (44/95), 34 (2/95), 34–35 (44/95), and 35–36 (5/95). The tooth position distribution of the mental foramen in different sexes was not statistically significant.

### Lower lingual canal

By tracing the LCS on the axial plane in the 3D plane, the anastomosis of the LCS and MC can be confirmed in combination with the sagittal and coronal planes (Fig. 4). It is further proved that the LCS anastomoses with the MC. Using this technique, these anastomoses in the mandible are easy to observe.

The observational sample size of the LCS was 95 CBCTs. In this test, the occurrence rate of the lingual foramen below the mandible was 95/95, 75/95 on the left side, and 77/95 on the right. A total of 229 LCS were detected, of which 58 were connected to the MC and anterior extension ducts such as the mental foramen or anterior annulus. Since the LCS is tubular in the mandible, the opening position of the LCS on the mandible was recorded. When it was found to coincide with the MC and its extension canal, the anastomosis was recorded together. The position of the corresponding tooth is illustrated in Fig. 4. When the LCS was anastomosed on the mandible, the corresponding tooth positions when the pipe is opened were 45 (4/95), 46 (18/95), 47 (2/95), 31 (1/95), 33 (1/95), 34 (2/95), 35 (13/95), and 36 (17/95), and the corresponding teeth during pipeline anastomosis were 44 (5/95), 45 (17/95), 46 (1/95), 47 (1/95), 31 (1/95), 32 (1/95), 33 (2/95), 34 (11/95), 35 (16/95), and 36 (3/95). Among them, an anastomosis of mandibular lingual foramen in two different tooth positions with the MC occurred in one individual on the right side and four on the left side. Therefore, the anastomosis rate of the right lingual foramen was 23/95 and that of the left side was 30/95. The anastomosis of the left mandibular lingual foramen in all samples was in front of the mental foramen, and both were in the caudal direction. The anastomosis of the right mandibular lingual foramen was all below the mental foramen, with 21/229, 1/229, and 2/229 in front, distal, and within 2.5 mm of the mental foramen, respectively.

### The bony canal at the apex of the tooth

BCAT occurs in the left and right sides of canines, premolars, and molars. The appearance rate, diameter, and coincidence rate of the BCAT at the DVR and 3D levels in the appeal tooth area are recorded in Table 2. Due to the inclusion criteria (Fig. 1), the sample size between the left and right sides was different. The occurrence rates of different types of BCAT in the canine, premolar, and molar regions are recorded in Table 4. The occurrence rate of bone canals at different ages was statistically significant (*p* < 0.001). The appearance of bone canals in different sexes was not statistically significant (*p* = 0.513).

**Table 4:** Frequency of the BCAT

	47	46	45	44	43	33	34	35	36	37
Type 1 occurrence rate of DVR	91.7	71.1	50	50	67.9	64.3	51.9	34.5	60	53.3
(95% CI)	(73.3, 110)	(55.9, 86.2)	(31.7, 68.3)	(28.4, 71.6)	(49.4, 86.3)	(45.4, 83.2)	(31.7, 72)	(16.1, 52.9)	(41.4, 78.6)	(24.7, 81.9)
Type 2 occurrence rate of DVR	8.3	13.2	15.6	20.8	32.1	35.7	33.3	34.5	20	6.7
(95% CI)	(-10, 26.7)	(1.9, 24.4)	(2.3, 28.9)	(3.3, 38.4)	(13.7, 50.6)	(16.8, 54.6)	(14.3, 52.3)	(16.1, 52.9)	(4.8, 35.2)	(-7.6, 21)
Type 3 occurrence rate of DVR	0	15.8	34.4	20.8	0	0	14.8	31	20	40
(95% CI)	(0, 0)	(3.6, 27.9)	(17, 51.8)	(3.3, 38.4)	(0, 0)	(0, 0)	(0.5, 29.1)	(13.1, 48.9)	(4.8, 35.2)	(11.9, 68.1)
Type 1 occurrence rate of 3D	100	55.3	56.3	58.3	75	67.9	59.3	44.8	40	53.3
(95% CI)	(100, 100)	(38.7, 71.8)	(38.1, 74.4)	(37.1, 79.6)	(57.9, 92.1)	(49.4, 86.3)	(39.5, 79.1)	(25.6, 64.1)	(21.4, 58.6)	(24.7, 81.9)
Type 2 occurrence rate of 3D	0	39.5	25	37.5	25	32.1	33.3	41.4	40	20
(95% CI)	(0, 0)	(23.2, 55.8)	(9.1, 40.9)	(16.6, 58.4)	(7.9, 42.1)	(13.7, 50.6)	(14.3, 52.3)	(22.3, 60.4)	(21.4, 58.6)	(-2.9, 42.9)
Type 3 occurrence rate of 3D	0	5.3	18.8	4.2	0	0	7.4	13.8	20	26.7
(95% CI)	(0, 0)	(-2.2, 12.7)	(4.5, 33)	(-4.5, 12.8)	(0, 0)	(0, 0)	(-3.2, 18)	(0.4, 27.1)	(4.8, 35.2)	(1.3, 52)

*CI *Confidence interval, *BCAT *Bony canal at the apex of the tooth, *DVR *Dental volume reformat, *3D *Three-dimensional. The sample size is 81 on the right and 80 on the left

## Discussion

Existing studies have proved that the MC, mental foramen, and lingual canal are anatomical structures that should be carefully evaluated before oral treatments, such as mandible surgeries, root canal treatments, apical surgeries, and tooth extractions. In this retrospective study, we aimed to further improve the understanding of the important anatomical structures in the mandible by jointly studying the MC, MF, LCS, and BCAT.

### Mandibular canal and mental foramen

Previous autopsy and CBCT studies have demonstrated that mandibular incisor tubes occur commonly; however, most extend between the first premolars and fangs and rarely reach the area below the central incisors [7, 8]. This occurs owing to the large difference in the position of the third molar within the mandible. Therefore, this study measured the MC and its extension, from the second molar region to the cusp area. Additionally, the anatomical parameters of the MC recorded the observation angle in the long axis of the tooth, and this angle is more conducive to measuring the distance between the MC and the tooth root. Mardinger et al. reported that the diameter range of the mandibular incisor tube was 0.48–2.9 mm. When the tube wall was unclear, the measurement data on the mandibular duct and its extension pipe were recorded as 0 in this study; therefore, the diameter of the head and tail directions of the tube in the cusp area were as follows: 0–2.34 mm on the right and 0–1.54 mm on the left, where the minimum recorded values were 0.45 mm and 0.55 mm on the right and left, respectively. The diameter range was 0.45–2.34 mm, which is close to the measurements reported by Mardinger. According to Hur et al. [9], the diameter in the first and second molars were 3.31 ± 0.8 and 3.1 ± 0.6 mm (range 2.1, 4.9 mm, and 2.4, 4.3 mm), respectively. In this study, the diameter in the first and second molars on the right was 2.40 ± 0.45 mm and 2.70 ± 0.55 mm, respectively, and 2.51 ± 0.44 mm and 2.69 ± 0.48 mm, respectively on the left.

### Lower lingual canal

Mandibular lingual foramen anomalies can lead to the death of the patient. Katakami et al. [6] reported the anastomosis rate of the lower lingual canal and MC and its extension canals, such as the mental foramen and anterior ring, as follows: the anastomosis rate of the first premolar (1/155) and second premolar (24/155), the first molar (5/155), the third molar (1/155), and no anastomosis of the lingual canal with the MC was observed in the rest of the teeth. According to the corresponding tooth position of the LCS opening in each tooth position recorded on the mandible, there were anastomotic central incisors (1/229), canines (1/229), first premolars (2/229), second premolars (17/229), first molars (35/229), and second molars (2/229), and no anastomosis was observed for the rest of the teeth. Additionally, this study found that the anastomosis of the LCS and the MC can be tracked more easily and accurately through the method illustrated in Fig. 4. Therefore, we can quickly and accurately observe the opening position and anastomotic position of LCS and MC when we use the conventional method to observe the distribution of the LCS, as illustrated in Fig. 4, and further avoid the LCS damage to the side tubes.

### The bony canal at the apex of the tooth

According to the observation of this experimental group, the BCAT is a common anatomical structure in the mandible; however, there are few CBCT imaging studies on the BCAT. In this study, the MC and its surrounding canals were determined. Simultaneously, to verify our ideas, we observed the BCAT from multiple angles through DVR and 3D layers and adjusted the viewing angle to verify the root canal and the authenticity of the presence of the BCAT. From Table 4, it can be observed that the BACT not only has a high occurrence rate in the canine area, premolar area, and molar areas but also connects with the MC. Owing to the differences in viewing angles at the 3D and DVR levels, the connection recorded in this test is direct or has obvious evidence of a connection. Wilbrand et al. [10] and Lindgre et al. [11] reported that calcium hydroxide enters the MC during root canal treatment, and a series of reactions lead to skin damage. Among them, the root tip of the former affected tooth has a significant distance from the MC. Hur et al. [9] reported that the MC has branches connected with the roots of the mandibular teeth. We previously suspected that the subapical bone canal may be the branch of the MC going to the apex of the tooth root; however, some of the BACT have a larger diameter, and the BACT is connected to the root tip of the tooth but not to the MC. The connection rate of pipes did not exceed 53%, without sufficient evidence to prove the appeal conjecture. Further research with imaging equipment such as magnetic resonance imaging, which facilitates visualization of soft tissues, may be required.

In this study, CBCT was utilized to examine the anatomical structure of the mandible from various angles. This approach enhanced our understanding of the mandibular anatomy. Additionally, it revealed the significance of unconventional observation methods in identifying frequently overlooked structures. The findings of this experiment emphasize the need for careful observation and identification of the MC and LCS prior to mandibular surgery, implant surgery, and root canal treatment. Moreover, the discovery of BACT holds promise in advancing our comprehension of the mandibular canal. This knowledge can potentially contribute to the development of AI-assisted diagnosis for jaw anatomy in the future.

## Conclusion

CBCT imaging can provide valuable assistance to surgeons in visualizing the anatomical structures within the mandible. By utilizing CBCT observation from various angles, surgeons can gain a clearer understanding of the precise location of important anatomical structures within the mandible. Advanced imaging techniques for preoperative planning contribute to improved safety and efficacy in dental interventions. Insufficient knowledge of mandibular anatomy prior to surgery significantly increases the risk of damaging crucial structures such as the mandibular neural canal, mental foramen, mandibular lingual canal, and subapical canal, which can lead to complications such as numbness and bleeding. Therefore, it is highly recommended that surgeons thoroughly examine CBCT scans from multiple angles prior to surgery to accurately identify and comprehend the location and characteristics of relevant anatomical structures within the mandible, thus minimizing the risk of intraoperative damage.

### Supplementary Information

**Supplementary Material 1.**

## Data Availability

The data presented in this study are available on request from the corresponding author.

## Abbreviations

3D: Three-dimensional
BACT: Bony canal at the apex of the tooth
CBCT: Cone-beam computed tomography
CI: Confidence interval
DVR: Dental volume reformat
LCS: Lingual canals
MC: Mandibular nerve canal
MF: Mental foramen

## References

[1] Bernardi S, Bianchi S, Continenza MA, Macchiarelli G (2017). Frequency and anatomical features of the mandibular lingual foramina: systematic review and meta-analysis. Surg Radiol Anat.

[2] Liao Q, Chen Y, Liao Y, Lian K (2022). Evaluation of the distribution characteristics of the mandibular lingual foramen and its potential risks during implant surgery using cone-beam computed tomography a cross-sectional, retrospective study. Clin Implant Dent Relat Res.

[3] Uchida Y, Yamashita Y, Goto M, Hanihara T (2007). Measurement of anterior loop length for the mandibular canal and diameter of the mandibular incisive canal to avoid nerve damage when installing endosseous implants in the interforaminal region. J Oral Maxillofac Surg.

[4] Budihardja AS, Pytlik C, Haarmann S, Holzle F (2006). Hemorrhage in the floor of the mouth after second-stage surgery: case report. Implant Dent.

[5] Dubois L, de Lange J, Baas E, Van Ingen J (2010). Excessive bleeding in the floor of the mouth after endosseus implant placement: a report of two cases. Int J Oral Maxillofac Surg.

[6] Katakami K, Mishima A, Kuribayashi A, Shimoda S, Hamada Y, Kobayashi K (2009). Anatomical characteristics of the mandibular lingual foramina observed on limited cone-beam CT images. Clin Oral Implants Res.

[7] Mardinger O, Chaushu G, Arensburg B, Taicher S, Kaffe I (2000). Anatomic and radiologic course of the mandibular incisive canal. Surg Radiol Anat.

[8] Sahman H, Sekerci AE, Sisman Y, Payveren M (2014) Assessment of the visibility and characteristics of the mandibular incisive canal: cone beam computed tomography versus panoramic radiography. Int J Oral Maxillofac Implants 29: 71–78. 10.11607/jomi.3304 10.11607/jomi.3304 24451856

[9] Hur MS, Kim HC, Won SY (2013). Topography and spatial fascicular arrangement of the human inferior alveolar nerve. Clin Implant Dent Relat Res.

[10] Wilbrand JF, Wilbrand M, Schaaf H, Howaldt HP, Malik CY, Streckbein P (2011). Embolia cutis medicamentosa (Nicolau syndrome) after endodontic treatment: a case report. J Endod.

[11] Lindgren P, Eriksson KF, Ringberg A (2002). Severe facial ischemia after endodontic treatment. J Oral Maxillofac Surg.

