# Supplementary material for: Study of the mandibular canal and its surrounding canals by multi-view cone-beam computed tomography
Source: Insights Imaging. 2024 Apr 8;15:103. doi: 10.1186/s13244-024-01676-x (PMC11001830; doi:10.1186/s13244-024-01676-x)

Study of the mandibular canal and its surrounding canals by multi-view cone-beam computed tomography

ELECTRONIC SUPPLEMENTARY MATERIAL

Figure 2-A Legends

This is the original image of part A in Figure 2, which includes axial, sagittal and panoramic images at the same level.

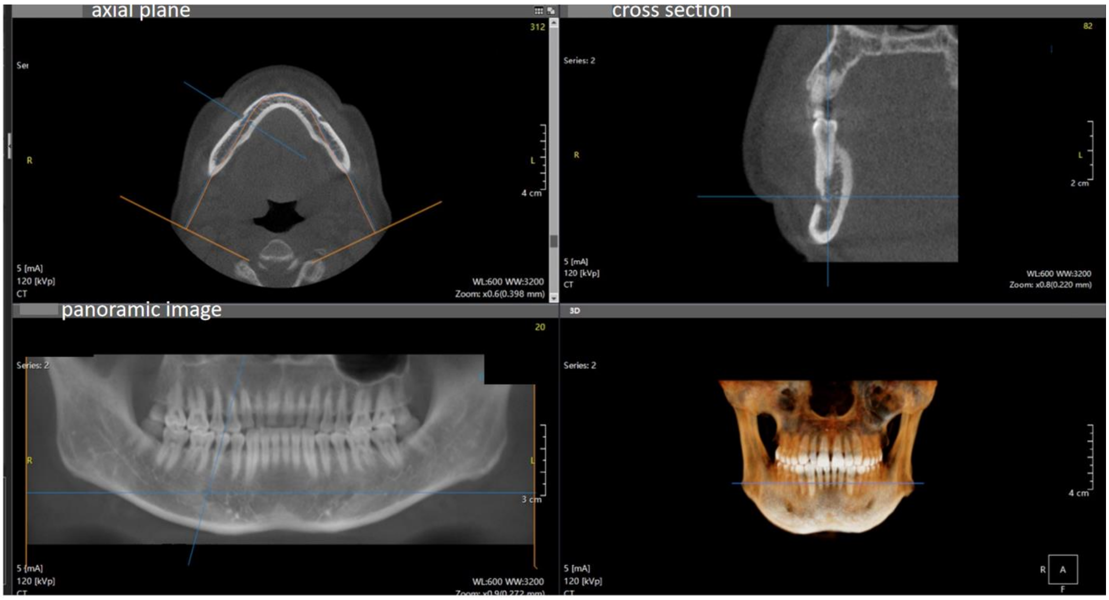

Figure 2-B Legends

This is the original image of part B in Figure 2, which includes axial, sagittal and panoramic images at the same level.

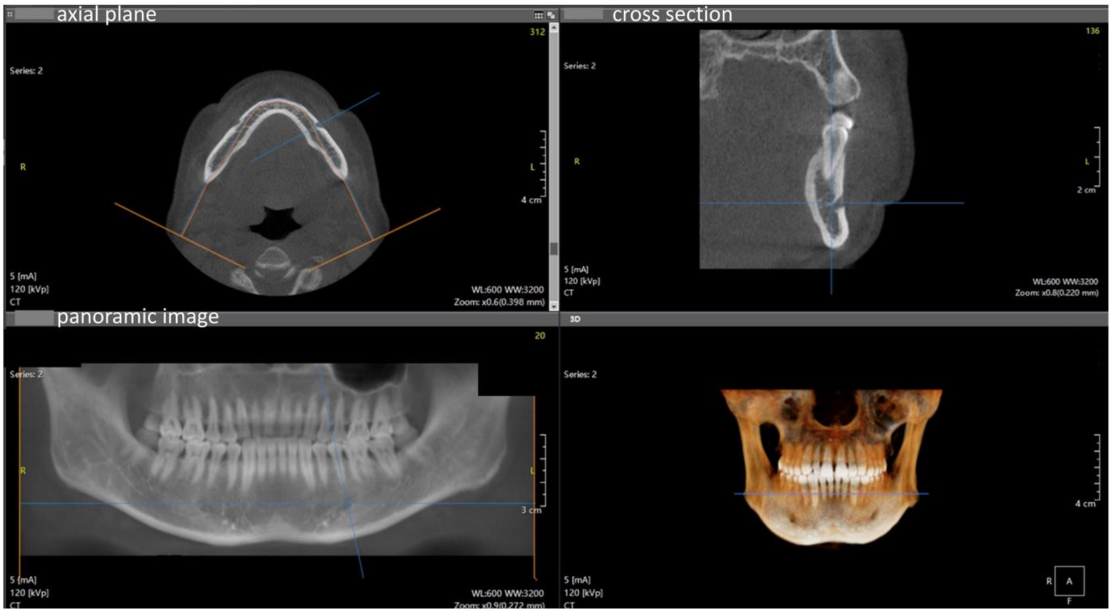

Figure3-A-1/2 Legends

This is the original image of part A（1-2） in Figure 3, which includes axial, sagittal and panoramic images at the same level.

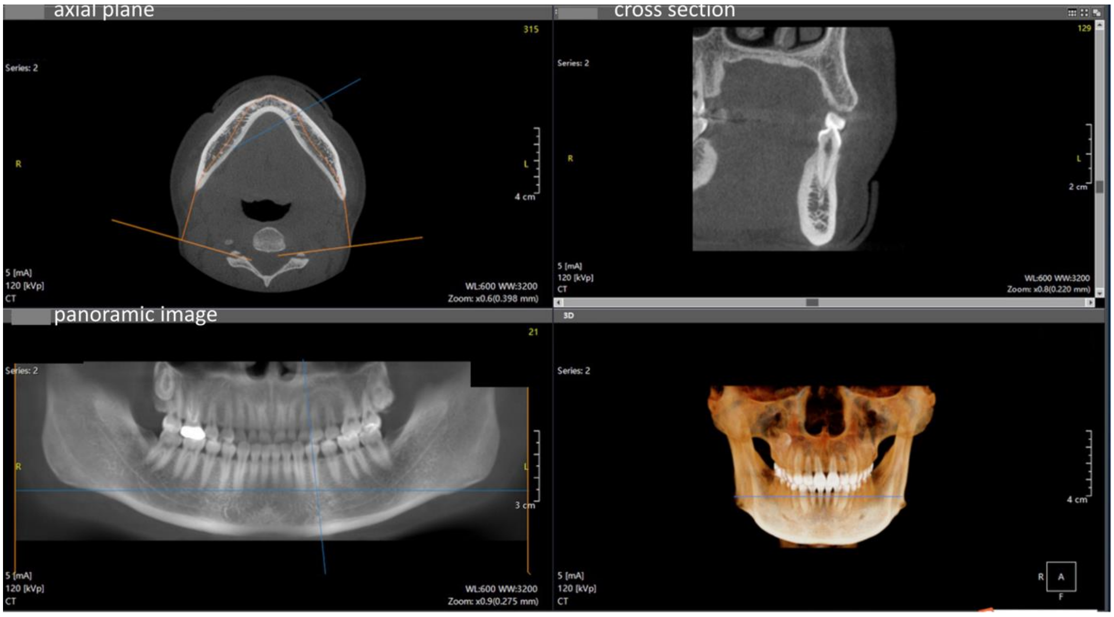

Figure3-A-3/4 Legends

This is the original image of part A (3-4) in Figure 3, which includes axial, sagittal and coronal images at the same level.

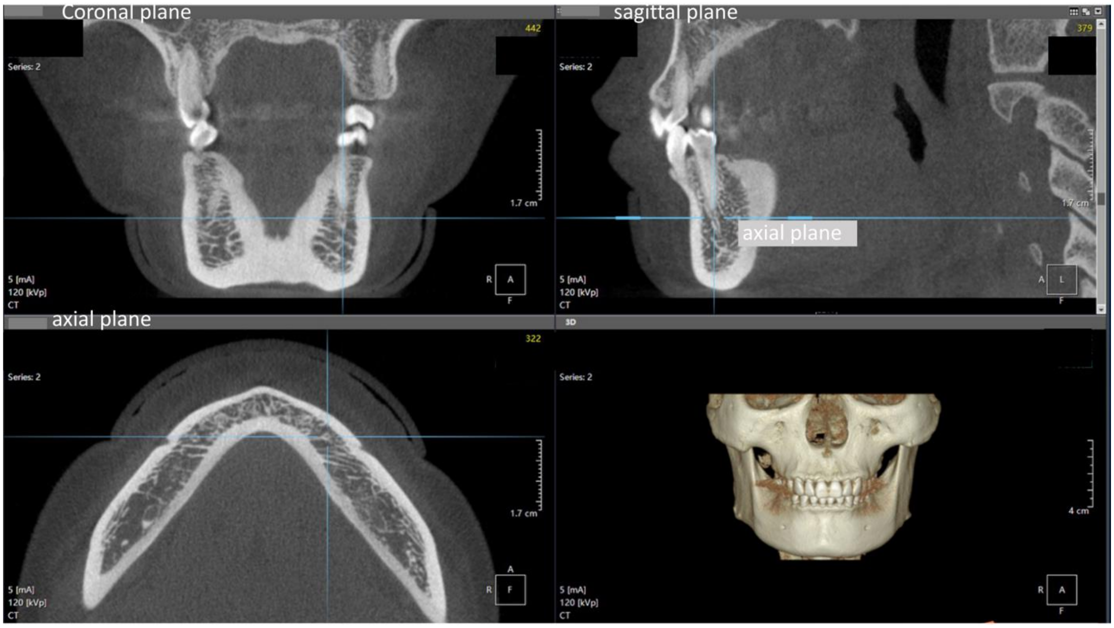

Figure3-A-5/6 Legends

This is the original image of part A (5-6) in Figure 3, which includes axial, sagittal and coronal images at the same level.

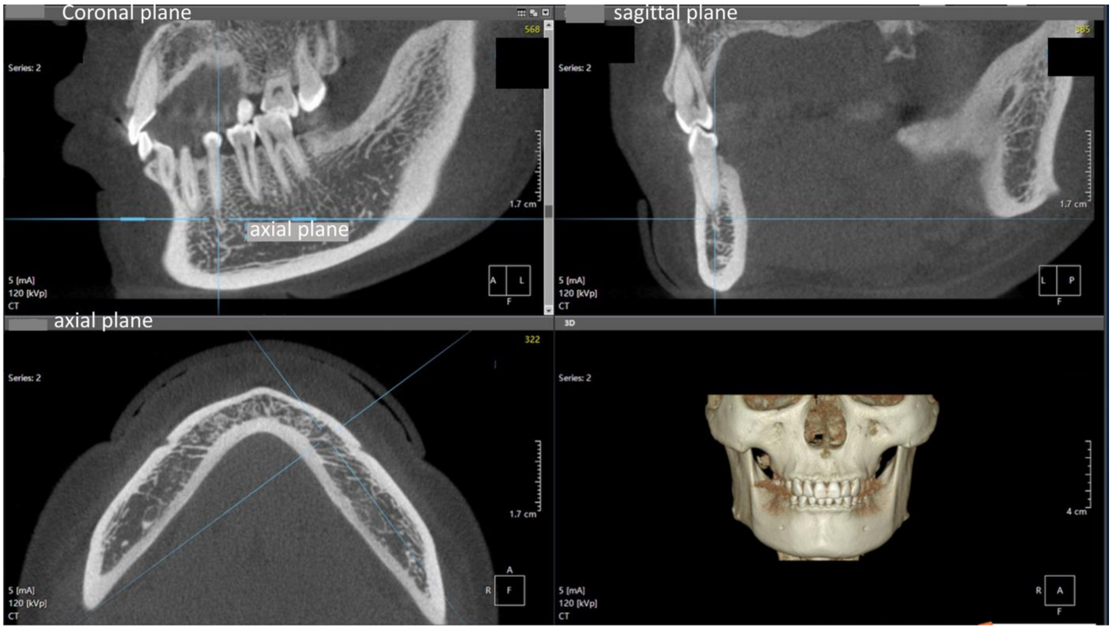

Figure3-B-1/2 Legends

This is the original image of part B（1-2） in Figure 3, which includes axial, sagittal and panoramic images at the same level.

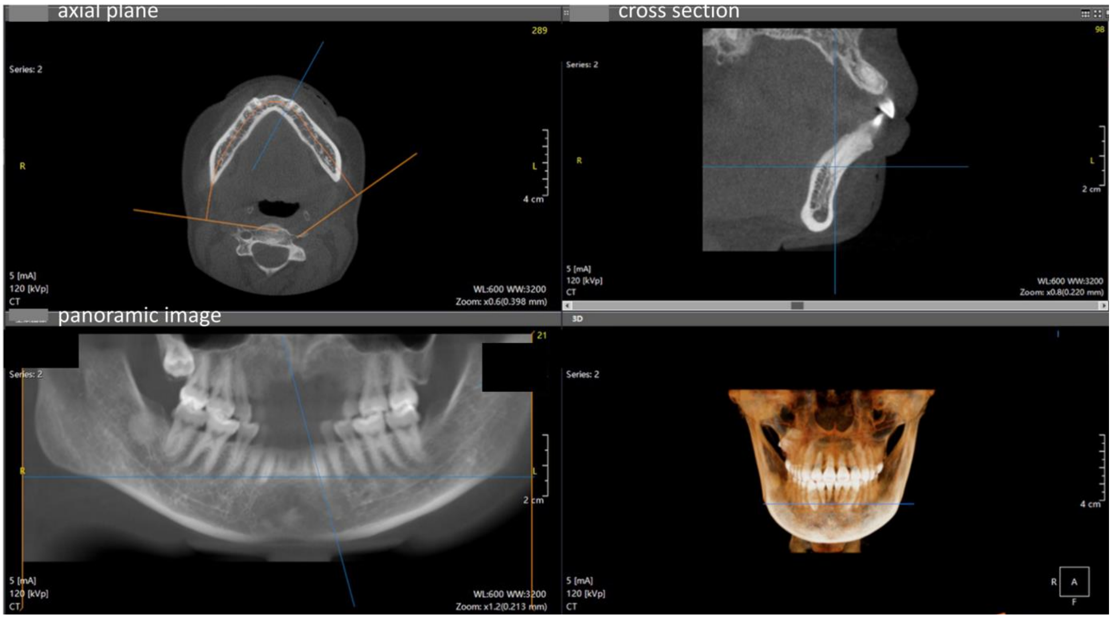

Figure3-B-3/4 Legends

This is the original image of part B (3-4) in Figure 3, which includes axial, sagittal and coronal images at the same level.

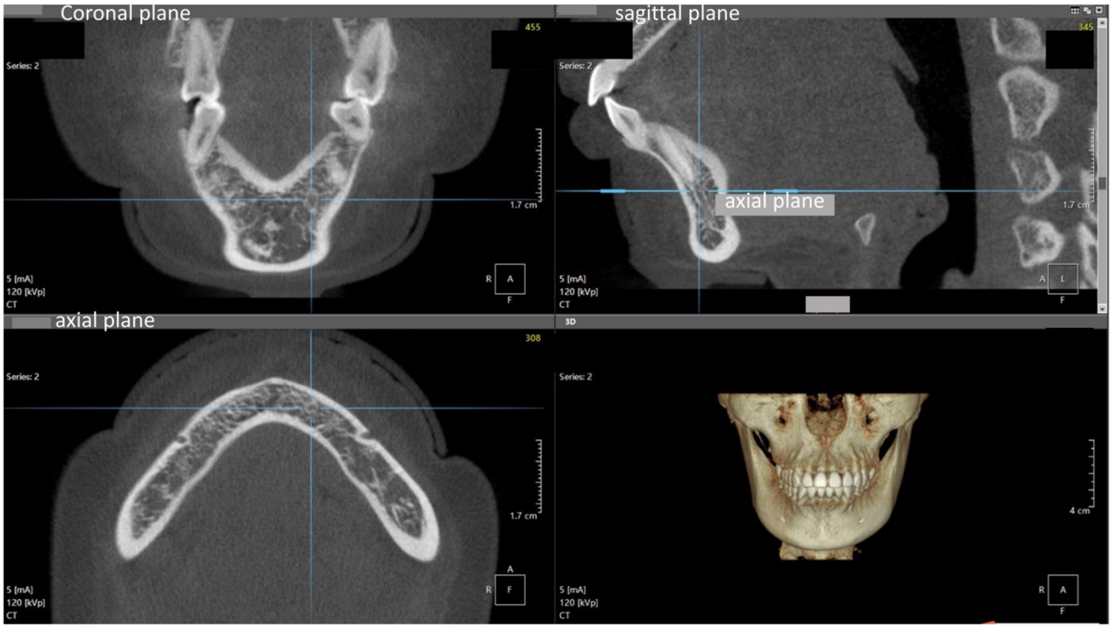

Figure3-B-5/6 Legends

This is the original image of part B(5-6) in Figure 3, which includes axial, sagittal and coronal images at the same level.

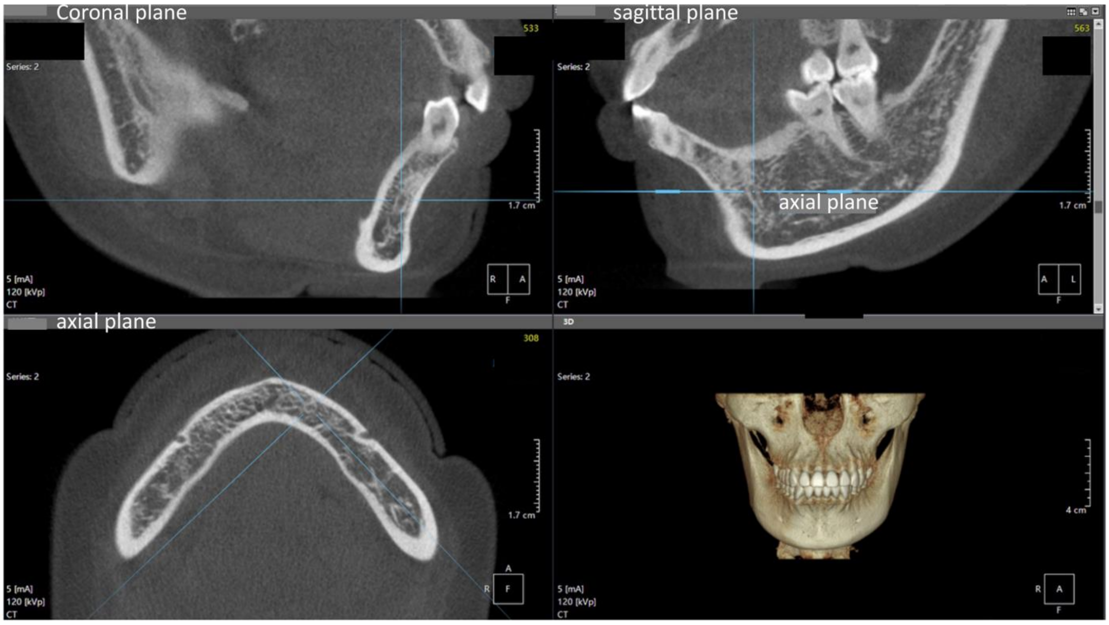

Figure3-C-1/2 Legends

This is the original image of part C（1-2） in Figure 3, which includes axial, sagittal and panoramic images at the same level.

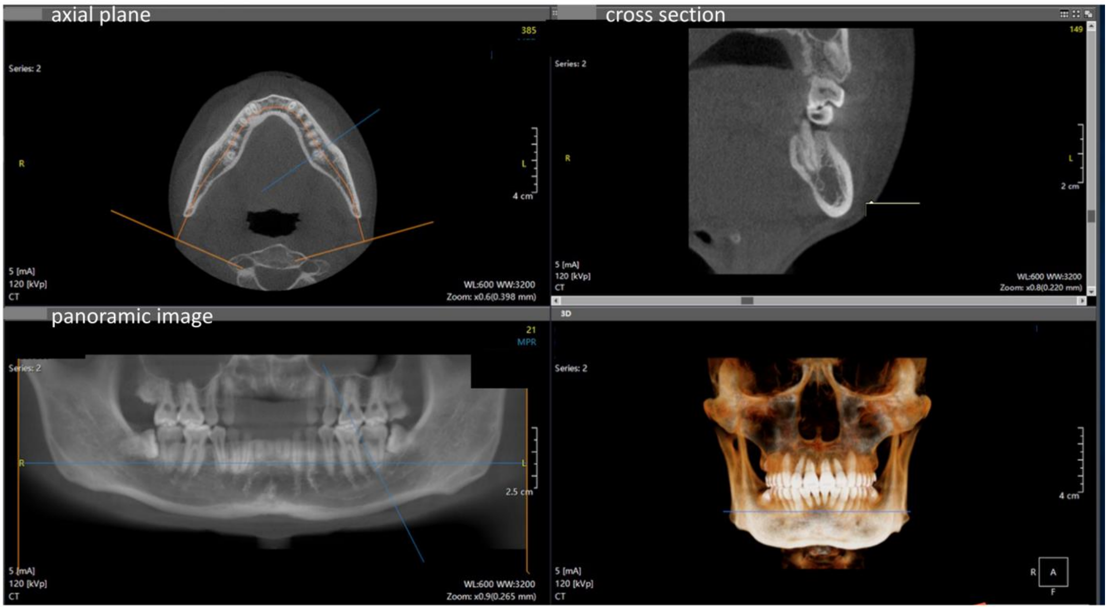

Figure3-C-3/4 Legends

This is the original image of part C(3-4) in Figure 3, which includes axial, sagittal and coronal images at the same level.

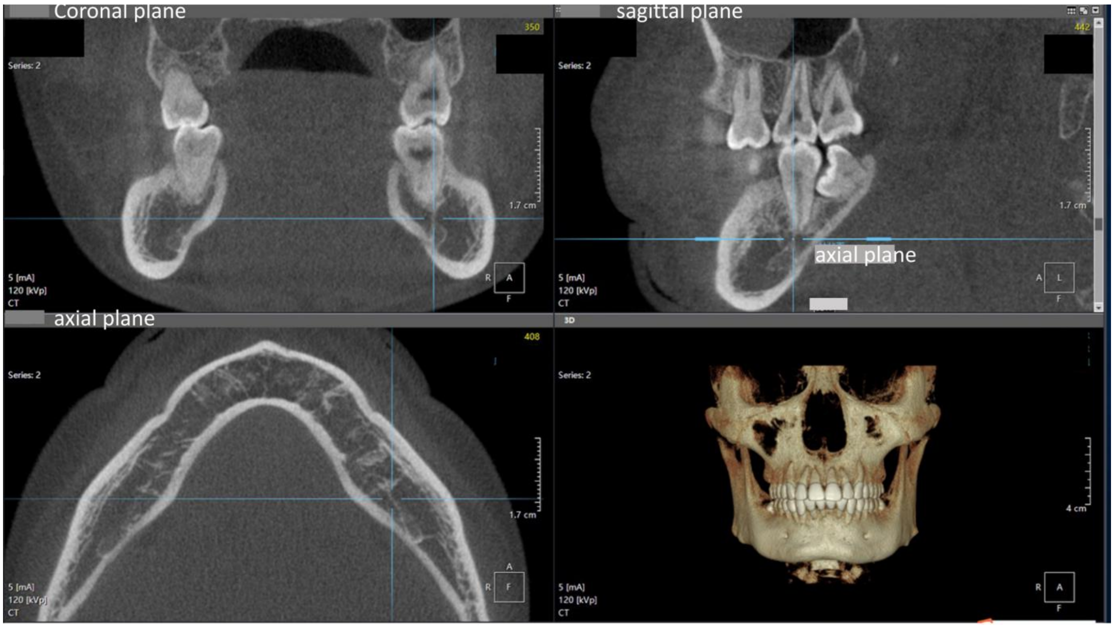

Figure3-C-5/6 Legends

This is the original image of part C（5-6） in Figure 3, which includes axial, sagittal and coronal images at the same level.

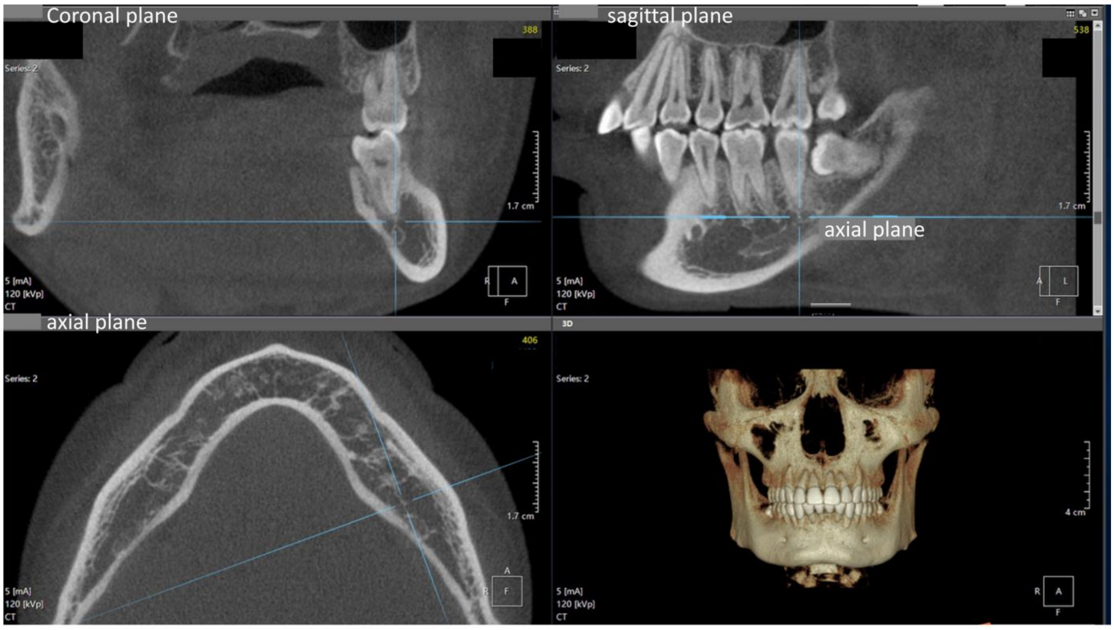

Figure4-A Legends

This is the original image of part A in Figure 4, which includes axial, sagittal and coronal images at the same level.

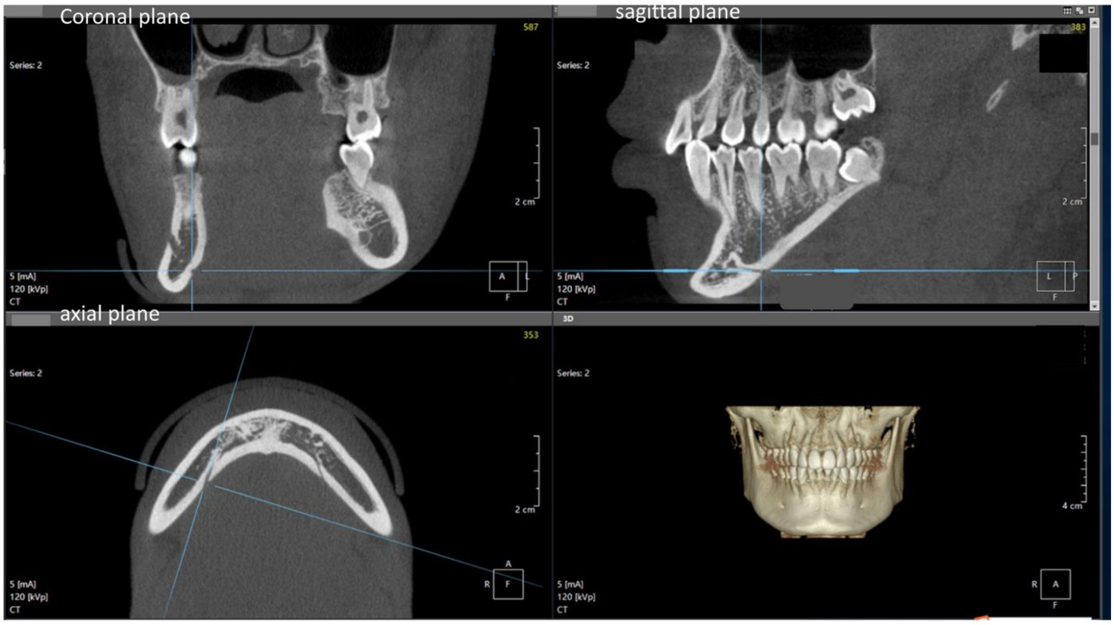

Figure4-B Legends

This is the original image of part B in Figure 4, which includes axial, sagittal and coronal images at the same level.

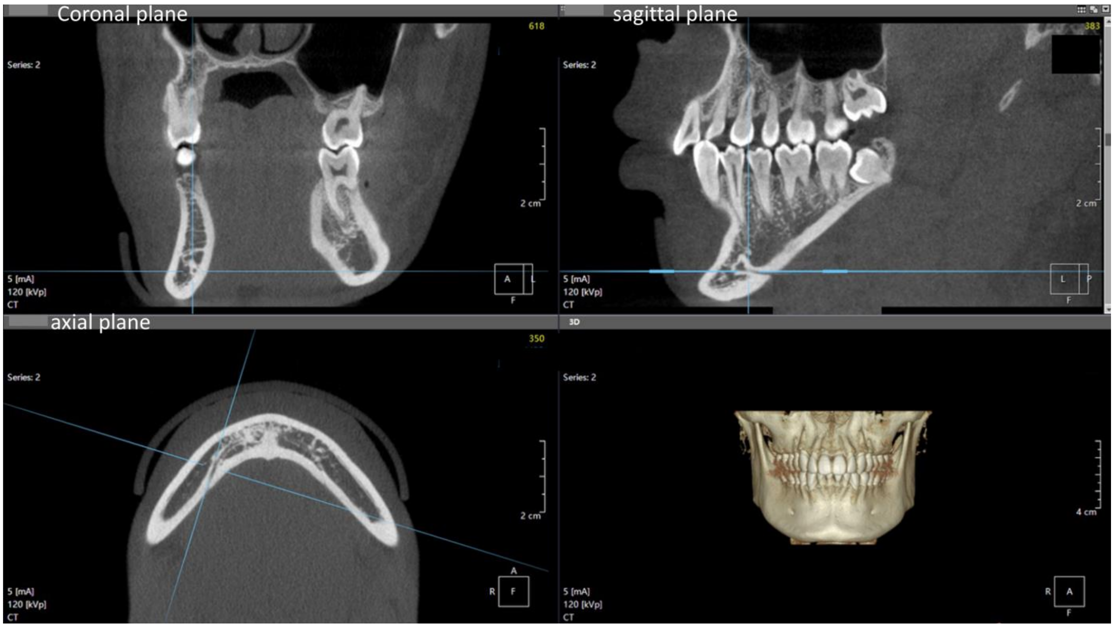

Figure4-C Legends

This is the original image of part C in Figure 4, which includes axial, sagittal and coronal images at the same level.

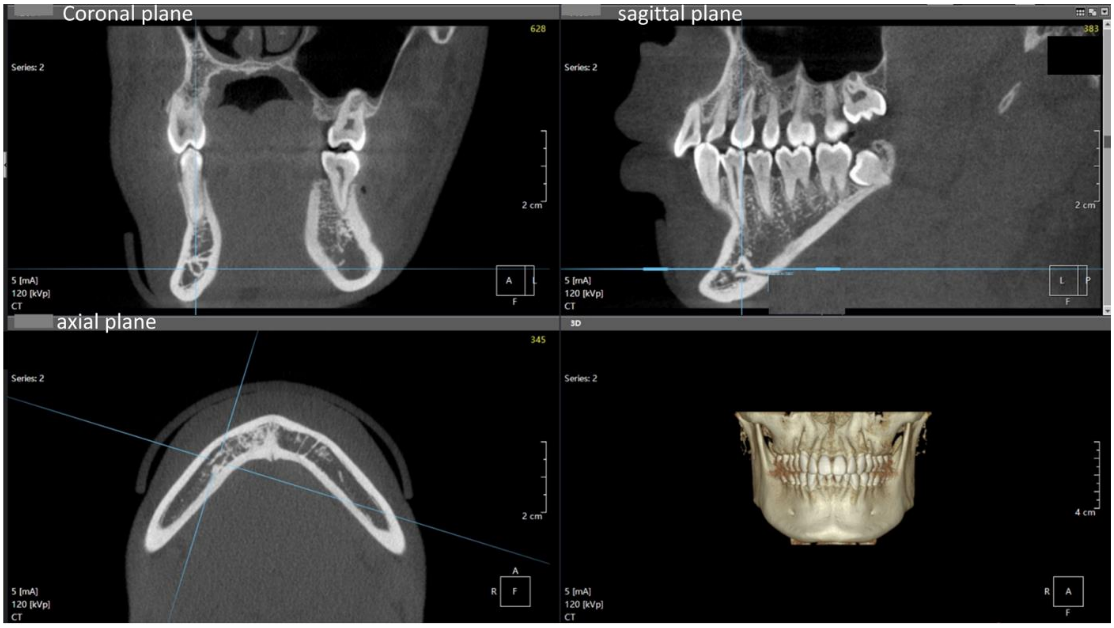

Supplement: Supplementary file 1 — Supplementary Material 1. [file 13244_2024_1676_MOESM1_ESM.pdf]
